# Guidelines for collection and processing of lungs from aged mice for histological studies

**DOI:** 10.1080/20010001.2017.1313676

**Published:** 2017-04-21

**Authors:** John Morton, Timothy A. Snider

**Affiliations:** ^a^ Department of Comparative Medicine, School of Medicine, University of Washington, Seattle, WA, USA; ^b^ Department of Veterinary Pathobiology, College of Veterinary Medicine, Oklahoma State University, Stillwater, OK, USA

**Keywords:** Aged mice, lungs, geropathology, lung collection protocol, histology

## Abstract

Pulmonary inflammation and the development of spontaneous lung tumors are important age-related lesions in mice. Therefore, gross and histological examination of the respiratory system is a critical component of geropathology research studies for translating surrogate endpoints to clinical aging studies. Collection, trimming, and processing of lung tissue from aged mice require a high-quality sequential process since aged mice are irreplaceable resource-intensive animal models. This protocol provides a basic technique that provides excellent sections for histological evaluation of the respiratory system of old mice suitable for most research applications. The points of emphasis are infusing the lungs at necropsy with formalin through the airways to prevent atelectasis artifacts that can preclude accurate histological evaluation, and embedding of anatomically oriented *in toto* lung lobes to allow for complete and thorough evaluation of all lung regions.

## Introduction

Aged mice in preclinical aging studies represent an investment of extensive amounts of time, resources, and effort, and generally cannot be replaced. Geropathology assessment at the end of the study is a key element in helping to define the pathological basis of aging endpoints [1] and in testing the efficacy of interventions. Diseases of the respiratory system in mice represent a significant lesion burden with increasing age depending on the background strain. For example, pulmonary inflammation and spontaneous neoplasms are important age-related lesions in C57BL/6 and CB6F1 mice [2]. Accurate characterization of these lesions depends upon the proper collection and processing of pulmonary tissue for histological examination. Improperly collected and processed tissues would be detrimental to the study since lesion distribution and scoring could not be accurately assessed, resulting in useless or misleading information. In most instances, the collection of lung tissue is only one aspect of a detailed postmortem examination involving observation and collection of all major organ systems. For the purposes of this focused protocol, details are restricted to the collection and processing of trachea and lung tissue and are intended as a guideline, especially for those less familiar with the geropathology of old mice. It is a basic protocol that can be adjusted depending on the experimental needs of the project.

## Basic protocol 1: Collection of the lungs from old mice

### Equipment and materials

- Dissecting board with cork or rubber surface
- Spray bottle with 70% ethanol
- Pins (to tack down carcass, user’s preference)
- Forceps
- Scissors
- 3 ml syringe with 23 g needle
- 10% neutral buffered formalin (at least 25 ml to maintain recommended 15–20:1 volume:tissue ratio)
- Permanent marker for labeling
- Metric weight scale

### Protocol steps

**Figure 1.**
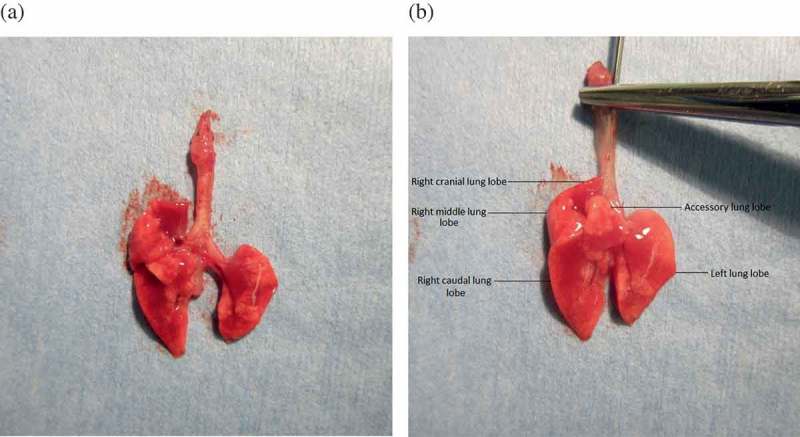
Lungs are gently inflated with formalin to provide the best structural format for assessing histopathology and are shown ventral side up: (a) uninflated lung tissue; (b) lung tissue inflated with formalin.

1. Prepare a container of 10% neutral buffered formalin, and label the container with all available identification parameters (the minimum database would include mouse identification number, mouse strain, weight, research study, and date).
2. If mice need to be euthanized, approved euthanasia procedures must be followed. Body weights are recorded.
3. The mouse carcass is secured to a dissecting board using tacks or pins. The mouse is positioned in dorsal recumbency with full extension of pelvic and pectoral limbs, and soaked with 70% ethanol using a spray bottle to keep hair out of the way while dissecting.
4. The skin over the sternum is grasped with forceps and an initial incision is made at the ventral midline. The incision is continued cranially and caudally along the ventral midline to extend from the chin to the pubis.
5. The skin is gently reflected bilaterally at the ventral midline incision extending from the ventral thorax to the abdomen, completely exposing the subcutis of the ventral and lateral thorax and abdomen.
6. A ventral midline incision is made into the abdomen to expose the peritoneal cavity and the xiphoid process.
7. The xiphoid process is grasped with forceps, and scissors are used to cut the ribs on each side of the sternum at about the mid-level of the body of the ribs. The ventral diaphragm muscle is cut with scissors or scalpel in an amount sufficient to release it from the rib portions to be removed. The xiphoid is retracted cranially as the ribs are cut, extending the cuts on both sides to the thoracic inlet. Subsequently, the rib cage is removed and the entire thoracic cavity exposed. Care should be taken not to damage the lungs while cutting, as this could prevent inflation later.
8. The tongue is removed by cutting the mandibular symphysis with scissors and retracting the tongue caudally with forceps.
9. While grasping the tongue with forceps and retracting caudally, all dorsal attachments are cut with scissors, removing the tongue, esophagus, trachea, heart, and lungs *en bloc*. The esophagus is the dorsal landmark through the cervical region. The aorta is the dorsal landmark in the thoracic cavity (i.e. the thoracic aorta is removed with the heart/lungs).
10. Heart and other organ tissues are separated from the trachea and lungs. Pulmonary tissue is examined grossly and findings are recorded.
11. A 23 g needle connected to a 3–5 ml syringe containing 10% formalin is gently threaded into trachea. The trachea is then clasped with forceps to hold the needle in place, and the lungs are gently inflated with formalin until they are fully expanded to a normal level as expected to fill the chest cavity (Figure 1). In general, there is no standard volume; however, under- and over-inflation should be avoided (see Commentary below).
12. The *en bloc* organ mass, including all lung lobes and trachea, is then placed in formalin. Lungs should be fixed for at least 24 h, although longer times may be required depending on specific stains or procedures.

### Commentary

Organ weights/lung weights can be obtained at any time before formalin infusions. The lungs can be removed from the *en bloc* sample by transection of the distal trachea just cranial to the tracheal bifurcation. Inflation of the lungs with formalin is a critical prequel to histological assessment. This step is often missed by the novice prosector. Atelectatic or under-inflated lungs impair evaluation of alveolar septa and can result in over-interpretation of interstitial pneumonia. Similarly, over-inflation of lungs can result in artifactual damage to septal walls and sometimes leads to an over-interpretation of emphysematous change. The volume of formalin necessary to properly inflate the lung tissue is variable, but is usually less than 1.5 ml. Typically, the amount of formalin delivered through the airways depends upon the estimation of expansion of the lung volume to physiological peak inspiration. To prevent over-inflation of the lungs, the infusion step can be completed with the lungs *in situ*, with formalin infusion stopping when the lung volume completely fills the thoracic cavity. More consistent airway infusion of the lungs can be obtained by slightly more complicated methods that typically require more time, equipment, and expertise. These are outlined by Braber et al. [3]. However, it should be noted that infusion of the lungs with formalin through the trachea with volume estimation at peak inspiration is acceptable and generally considered the best procedure. Depending upon the study questions, evaluation of the alveolar septa may be so critical that inflation of the lungs must be accurately consistent and/or alveolar septal capillaries must be cleared of remaining blood. To clear lungs of blood, formalin is injected into the right ventricle of the heart. The lungs will slightly inflate and transition from red/pink to completely white when this procedure is complete.

## Basic protocol 2: Post-fixation trimming of lung tissue for histological sectioning

### Equipment and materials

- Slotted, tissue cassettes
- Forceps
- Pencil or solvent-resistant marker or pen (to label tissue cassettes)
- 10% neutral buffered formalin

### Protocol steps

**Figure 2.**
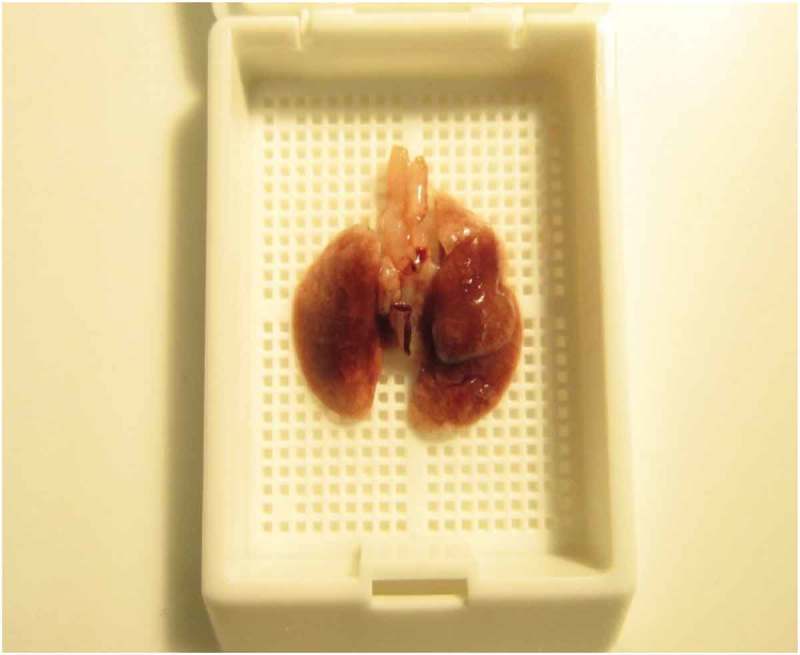
Lung tissue is placed in the cassette ventral side down to ensure consistent and uniform sectioning of all lung lobes for fixation to the glass slide.

**Figure 3.**
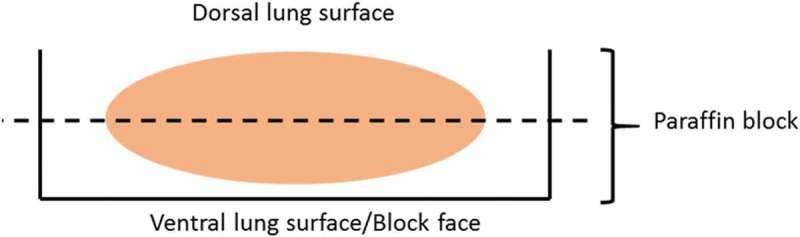
Mouse lung embedded *in toto*, ventral side down. Ventral lung represents the block face, which is ‘faced in’ via microtomy in the longitudinal horizontal (per figure orientation) direction until the appropriate cutting level is obtained, a level suggested and approximated by the dashed line.

1. The five lungs lobes are identified, anatomically oriented and placed *in toto* into a slotted tissue cassette, ventral side down (Figure 2).
  - left lung lobe
  - right cranial lung lobe
  - right middle lung lobe
  - right caudal lung lobe
  - accessory lung lobe.
2. During trimming, judgment calls are made as to whether grossly observed lesions would be missed from *in toto* sectioning. If so, lesions of concern are isolated (based upon gross observations), sectioned, and placed in the cassette. The remaining lung tissue is placed in the cassette and oriented (ventral side down) *in toto*.
3. The distal trachea and peritracheal connective tissue is placed flat in the same or a different cassette, depending on available space.
4. Following tissue processing and embedding of tissue (as placed in the cassette), the lungs can be sampled as appropriate to the particular study (Figure 3).

### Commentary

Post-fixation dissection and preparation of tissues for heat-processed, paraffin-embedded tissue sectioning is arguably one of the most important steps in the lung evaluation procedure. Standardized trimming of tissues is needed in research studies to maintain consistency in organ sampling across individual mice [4]. Notes and observations made during the gross examination should always be available and referenced during post-fixation dissection.

In most instances, the entire murine lung mass is amenable to histological evaluation *in toto. In toto* lung sectioning provides a complete look across hilar and peripheral lung regions for any possible lesions. In most cases, gross lesions observed during collection and preparation will be present following sectioning of *in toto* lungs. Exceptions include very large lesions that may not fit with the remainder of the lung and very small, often peripheral lesions that may be missed during tissue block face-in. For these reasons, some lesions (whether too big or too small) may need to be isolated and embedded individually. Inclusion of maximal pulmonary tissue from the entire murine lung mass on the glass slide is largely determined by the degree of block face-in before selection of the ideal tissue section to apply to the slide. Communication between the pathologist and histology technician is key, so that the technician can recognize the appropriate level of block face-in where maximal pulmonary tissue can be appreciated.

The standardized approach and protocol presented here enable the potential to exploit stereological analyses of the lungs from aged rodents. Stereological approaches, usually accompanied by mathematical and statistical computations, enable the analysis of shapes and measurements in three dimensions. This approach has the potential to minimize some bias encountered in the evaluation of histological sections in standard two dimensions. Its potential value in the surveillance of aging rodent lungs may reside in an area beyond the experimental objectives of the specific aging study, yet it still represents an attractive possibility. A thorough review of the potential applications in the lung is published elsewhere [5].

In summary, histological evaluation should include: (i) the *in toto* lung parenchyma, (ii) any grossly observed lesions that were separated from the *in toto* lung bloc, and (iii) the distal trachea with peritracheal/mediastinal connective tissue containing hilar oriented lymph nodes. It is critically important to include lymph nodes for histological evaluation as these are important for determining extension of neoplastic disease (metastasis) as a criterion for malignancy during the evaluation of lung tumors.

The Geropathology Research Network website (www.geropathology.org) has links on recommendations for organ sampling and trimming of other organ systems.
